# Loss of fibroblast growth factor 21 action induces insulin resistance, pancreatic islet hyperplasia and dysfunction in mice

**DOI:** 10.1038/cddis.2015.80

**Published:** 2015-03-26

**Authors:** W Y So, Q Cheng, A Xu, K S L Lam, P S Leung

**Affiliations:** 1 School of Biomedical Sciences, Faculty of Medicine, The Chinese University of Hong Kong, Hong Kong, China; 2 Department of Medicine, Li Ka Shing Faculty of Medicine, University of Hong Kong, Hong Kong, China

## Abstract

Fibroblast growth factor (FGF) 21 is an endocrine factor that normalizes glucose homeostasis and reduces insulin resistance in diabetes. Although the pancreas is an FGF21 target organ, its role in pancreatic islets remains obscure. This study aimed to elucidate the physiological role of FGF21 in pancreatic islets using FGF21-knockout (FGF21-KO) mice. Twenty-four-week-old male global FGF21-KO mice were used in this study. Glucose and insulin tolerance were assessed. Expression of genes and proteins related to islet function and underlying mechanisms were also examined. Islet morphology and insulin-secreting capacity were further evaluated. FGF21-KO mice exhibited insulin resistance while being normoglycemic, associated with increases in beta-cell proliferation and insulin synthesis, acting as compensatory responses. This phenotype probably results from enhanced growth hormone (GH) sensitivity in FGF21-KO mouse islets. In addition, *ex vivo* FGF21 treatment in normal C57BL/6J mouse islets reduced GH signaling, probably via upregulation of peroxisome proliferator-activated receptor gamma (PPAR*γ*) and cytokine-inducible SH-2 containing (CIS) protein, whereas KO mouse islets displayed reduced PPAR*γ* and CIS expression. FGF21 treatment also reversed GH-induced insulin expression, beta-cell proliferation and GH-impaired glucose-stimulated insulin secretion (GSIS) in islets. Furthermore, distorted islet morphology and impaired GSIS were observed in KO mice, suggestive of islet dysfunction, whereas the enhanced insulin expression and impaired GSIS in FGF21-KO mouse islets could be reversed by blockade of GH signaling. Our data indicate that FGF21 is important in the regulation of beta-cell proliferation and insulin synthesis, probably via modulation of GH signaling. These findings provide evidence that FGF21 is an obligatory metabolic regulator in pancreatic islets and shed new light onto the role of endogenous FGF21 in the pathogenesis of insulin resistance and islet dysfunction.

Type 2 diabetes mellitus (T2DM), which is defined as hyperglycemia of sufficient magnitude to lead to detrimental effects, results when insulin resistance develops in association with dysregulated insulin secretion and loss of beta-cell mass.^1^ Insulin resistance prompts pancreatic islet compensatory responses such as increased beta-cell proliferation and insulin production. At this stage, whether an insulin-resistant individual will progress to frank hyperglycemia depends on the ability of islets to provide adequate compensatory insulin secretion.^2^

Fibroblast growth factor (FGF) 21 is an endocrine factor that belongs to the FGF family. It has been demonstrated to be a potent regulator of glycemia, lipid metabolism and energy homeostasis. FGF21 treatment reduces plasma levels of glucose and triglycerides, as well as improves insulin sensitivity and glucose clearance in diabetic mice; FGF21 protects rodents from weight gain and hepatosteatosis upon a high-fat diet challenge.^3, 4, 5^ FGF21 also improves lipoprotein profiles in nonhuman primates.^6^ Notably, pancreatic islets are one of the major FGF21 targets as FGF21 enhances beta-cell function and survival.^7^ In this context, our laboratory has recently defined the role of FGF21 in islet glucotoxicity under diabetic conditions.^8^

Growth hormone (GH) is synthesized and released by the anterior pituitary gland to regulate multiple physiological processes including growth and metabolism.^9, 10^ When GH binds to the GH receptor (GHR) on cell surface, janus kinase 2 (JAK2) is phosphorylated and activated; it in turn phosphorylates members of the signal transducers and activators of transcription proteins (STAT), mainly 5A and 5B, thus leading to their nuclear translocation to regulate target genes transcription.^9^ Several molecules have been identified to modulate GH signaling, including suppressors of cytokine signaling (SOCS), cytokine-inducible SH-2 containing (CIS) protein,^11, 12^ and peroxisome proliferator-activated receptor gamma (PPAR*γ*),^13^ which directly inhibit phosphorylation or transcriptional activity of JAK and STAT. It is well documented that GH antagonizes insulin action and induces *in vitro* and *in vivo* insulin resistance.^10^ Chronic exposure to GH modulates insulin signal transduction in muscle and adipose tissues^14, 15^ while causing hyperinsulinemia and insulin resistance.^16^ Clinically, humans with acromegaly or with infused GH exhibit reduced hepatic and extrahepatic insulin actions.^17, 18^ On the other hand, it has long been known that GH stimulates beta-cell proliferation and insulin synthesis.^19, 20, 21^ Hypersecretion of GH in rats with GH-secreting tumors results in increased insulin levels with beta-cell proliferation and the development of islet hyperplasia.^22, 23^ These findings suggest that GH acts on beta cells, leading to compensatory responses that cope with the increased insulin resistance.

Meanwhile, recent studies have found that FGF21 interacts with GH; transgenic mice with FGF21 overexpression show reduced growth and blunted hepatic GH signaling.^24^ Apart from effects on the liver, FGF21 also mediates the chronic undernutrition induction of GH insensitivity.^25^ Increased FGF21 expression during food restriction directly suppresses growth plate chondrocyte proliferation and differentiation, thus reducing skeletal growth.^26^ Notably, GH can stimulate hepatic FGF21 expression directly or indirectly, suggesting a negative feedback loop that prevents excessive GH signaling.^27, 28^ Although it is known that both FGF21 and GH are involved in the determination of insulin sensitivity and/or resistance, whether FGF21 modulates metabolic parameters via its interaction with GH has not been investigated. More importantly, it is well recognized that islet dysfunction has a critical role in controlling the progression of T2DM; however, the physiological role of FGF21 and the functional correlate between FGF21 and GH in the islets has not been explored.

In light of these findings and of the knowledge gap identified, this study aimed to elucidate the role of FGF21 in islet function and in the pathogenesis of T2DM and, in particular, to identify the role of GH signaling via the use of FGF21-knockout (KO) mice.

## Results

### FGF21-KO mice are normoglycemic, hyperinsulinemic and insulin resistant

**Figure 1:**
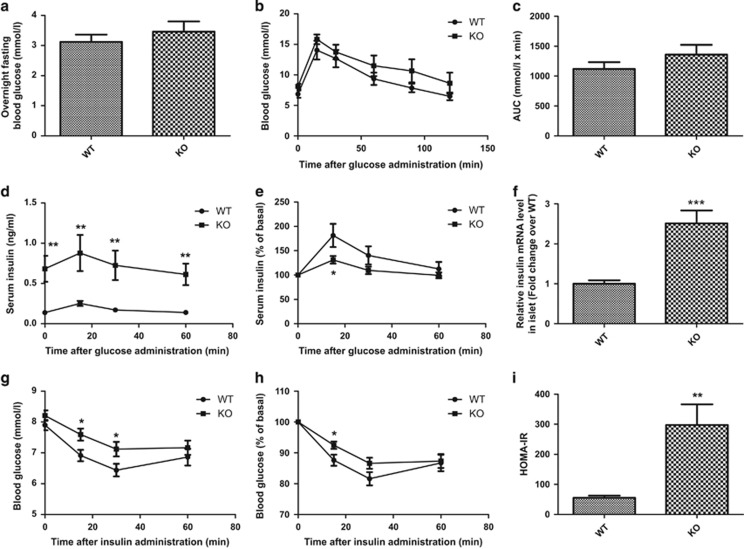
FGF21-KO mice are normoglycemic, hyperinsulinemic and insulin resistant. (**a**) Overnight fasting blood glucose. (**b**) Glucose tolerance by IPGTT, and (**c**) glucose profiles calculated from IPGTT as area under the curve (AUC). Serum insulin levels during IPGTT were expressed as (**d**) absolute values and (**e**) % of basal level. (**f**) Islet insulin mRNA levels (total RNA was extracted immediately after islet isolation). Blood glucose levels during ITT were expressed as (**g**) absolute values and (**h**) % of basal level. (**i**) Insulin sensitivity was indicated by HOMA-IR. *P<0.05, **P<0.01, ***P<0.001 *versus* WT (*n*=4–6 mice/batch; 4–6 batches). Data are mean±SE

FGF21-KO mice displayed similar overnight fasting blood glucose to values seen in wild-type (WT) mice (Figure 1a; WT=3.12±0.25 mmol/l; KO=3.46±0.34 mmol/l). FGF21-KO mice exhibited slightly higher glucose responses during intraperitoneal glucose tolerance test (IPGTT) to those in WT mice, but these were not significantly different (Figures 1b and c; area under the curve: WT=1119.84±112.56 mmol/l × min; KO=1358.51±164.50 mmol/l × min). Serum insulin levels of FGF21-KO mice were significantly elevated compared with WT mice during the glucose tolerance test (Figure 1d). However, the response to glucose, expressed as percent of basal insulin, was reduced in KO mice (Figure 1e). Islet insulin mRNA levels were upregulated in FGF21-KO mice (Figure 1f; ~2.5-fold greater than the WT mice). FGF21-KO mice were less insulin sensitive than WT mice with higher blood glucose concentrations at 15 min and 30 min after insulin injection (Figures 1g and h) and consistently, the ‘homeostasis model assessment' (HOMA-IR) index, which measures the degrees of insulin resistance, was also increased in FGF21-KO mice (Figure 1i).

### FGF21-KO mice display islet hyperplasia and increased beta-cell proliferation

**Figure 2:**
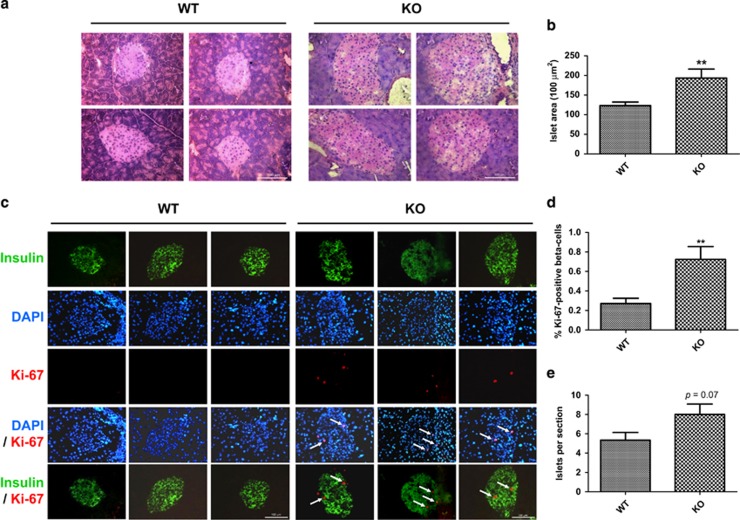
FGF21-KO mice display islet hyperplasia and increased beta-cell proliferation. (**a**) Representative photomicrographs of islets stained with hematoxylin and eosin, and (**b**) measurement of islet area. (**c**) Representative immunostaining of islets labeled for insulin (green), DAPI (blue) and Ki-67 (red). (**d**) Beta-cell proliferation was analyzed by counting the Ki-67-positive beta-cell number divided by total beta-cell number (% Ki-67-positive beta cells). (**e**) Islets per section. Scale bar=100 *μ*m. **P<0.01 *versus* WT (*n*=4–6 mice/batch; three batches). Data are mean±SE

FGF21-KO mouse islets were larger than those of WT mice (Figures 2a and b; WT=12311.36±884.54 *μ*m^2^; KO=19321±2290.51  *μ*m^2^), as shown by hematoxylin–eosin staining. FGF21-KO mice also had increased beta-cell proliferation (Figure 2c), as evidenced by immunostaining results. In islets from 24-week-old WT mice, the cell proliferation marker Ki-67 was virtually absent, but was easily detectable in FGF21-KO mice. Ki-67 staining was co-localized to the nuclei (labeled by DAPI) of beta cells (stained by insulin), indicating increased beta-cell proliferation (Figure 2d; WT=0.27±0.17; KO=0.72±0.39% Ki-67-positive beta cells). A slight, but statistically insignificant increase in islet number per pancreatic section was detected in FGF21-KO mouse islets (Figure 2e; WT=5.33±0.80; KO=8.01±1.08 islets per section). In addition, FGF21 deficiency did not affect islet apoptosis as shown in Supplementary Figure S1, suggesting that islet hyperplasia in FGF21-KO mice was mainly due to increased beta-cell proliferation.

### FGF21-KO mouse islets show distorted morphology and increased alpha-cell population

**Figure 3:**
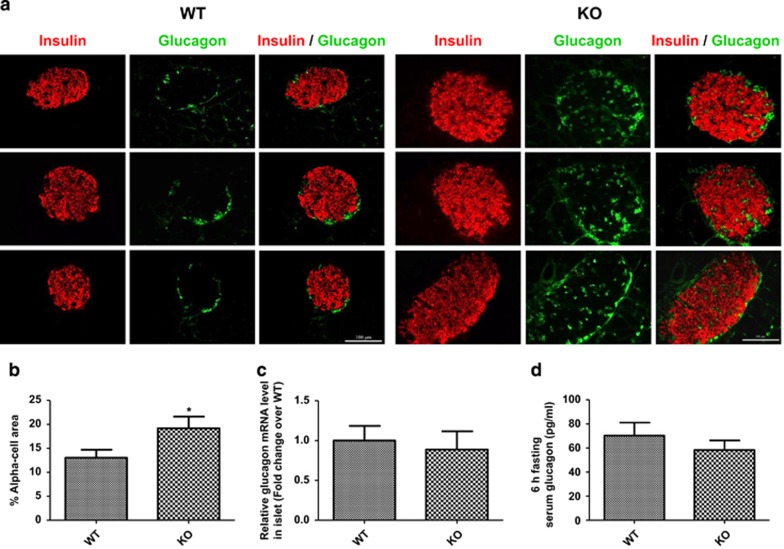
FGF21-KO mice show distorted islet morphology and increased alpha-cell population. (**a**) Representative photomicrographs of islets labeled for insulin (red) and glucagon (green). Scale bar=100 *μ*m. (**b**) % of alpha-cell area. (**c**) Islet mRNA and (**d**) 6-h fasting serum levels of glucagon. *P<0.05 *versus* WT (*n*=4–6 mice/batch; four batches). Data are mean±SE

Immunofluorescent staining of pancreatic islets from FGF21-KO mice revealed a distortion of islet cell distribution. In WT mouse islets, beta cells were located centrally, whereas alpha cells were distributed along the periphery of the islets. Unlike WT mice, some alpha cells in FGF21-KO mouse islets were found in the center of the islets (Figure 3a). No change was observed in delta-cell distribution as labeled by somatostatin (Supplementary Figure S2). In addition, FGF21-KO mouse islets exhibited an increase in alpha-cell area (Figure 3b; WT=13.05±1.67; KO=19.2±2.43% alpha-cell area), which is attributed by the increased alpha-cell proliferation as shown by Ki-67 staining (Supplementary Figure S3). However, no significant difference was found in islet mRNA or serum levels (after 6 h fasting) of glucagon between WT and FGF21-KO mice (Figures 3c and d).

### FGF21-KO mouse islets display enhanced GH sensitivity, whereas exogenous FGF21 treatments inhibit GH signaling in normal C57BL/6J mouse islets

**Figure 4:**
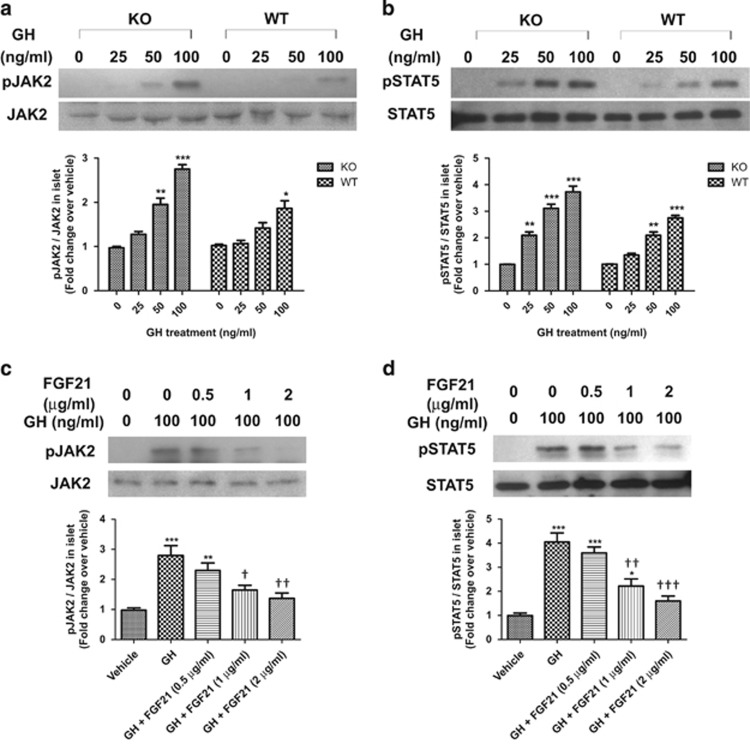
FGF21 inhibits GH signaling in islets. (**a**) Phosphorylated and total JAK2, and (**b**) phosphorylated and total STAT5 in FGF21-KO and WT mouse islets treated with GH for 15 min. *P<0.05, **P<0.01, ***P<0.001 *versus* 0 ng/ml (*n*=3–4). (**c**) Phosphorylated and total JAK2, and (**d**) phosphorylated and total STAT5 in normal mouse islets pre-treated with FGF21 (0–2 *μ*g/ml) for 72 h, followed by GH (100 ng/ml) treatment for 15 min. *P<0.05, **P<0.01, ***P<0.001 *versus* Vehicle; ^†^*P*<0.05, ^††^*P*<0.01, ^†††^*P*<0.001 *versus* GH (*n*=3). Data are mean±SE

To study the cross-talk between FGF21 and GH, serum GH levels in WT and FGF21-KO mice were measured in fed and overnight-fasted states. FGF21 deficiency had no effect on the production of GH in either fasting or fed state (Supplementary Figure S4A). GH action in islets was examined by determining GHR expression in both FGF21-KO and WT mouse islets. In 24-week-old mice, GHR mRNA was higher in FGF21-KO than WT mouse islets (Supplementary Figure S4B). However, significant change in GHR protein levels was not detected (Supplementary Figure S4C) while exogenous FGF21 treatment in normal mouse islets did not affect GHR expression (Supplementary Figures S4D and E). Isolated islet responses to GH were then studied by measuring JAK2 and STAT5 phosphorylation. After treatment with recombinant GH (0, 25, 50, or 100 ng/ml) for 15 min, FGF21-KO mouse islets showed enhanced phosphorylation of JAK2 and STAT5 compared with that in WT mouse islets (Figures 4a and b). To investigate the direct effects of FGF21 on GH action in islets, islets isolated from normal mice were pre-treated with recombinant FGF21 (0, 0.5, 1, or 2 *μ*g/ml) before GH-induced signaling was examined. After 72 h treatment, both JAK2 and STAT5 phosphorylation induced by GH (100 ng/ml) was reduced in a dose-dependent manner, in which the greatest effect was found at 2 *μ*g/ml of FGF21 (Figures 4c and d).

### GH-induced insulin expression and beta-cell proliferation are reversed by FGF21 in normal mouse islets

**Figure 5:**
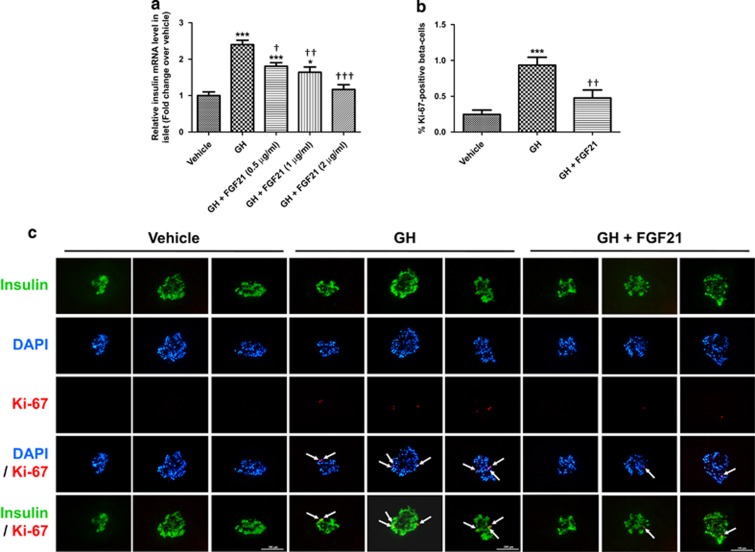
FGF21 reduces GH actions in islets at the functional levels. (**a**) Insulin mRNA levels in normal mouse islets treated with vehicle, GH (100 ng/ml), and with or without FGF21 (0.5–2 *μ*g/ml) for 72 h. *P<0.05, ***P<0.001 *versus* vehicle; ^†^*P*<0.05, ^††^*P*<0.01, ^†††^*P*<0.001 *versus* GH (*n*=4). (**b**) Normal mouse islets were treated with vehicle, GH (100 ng/ml), and with or without FGF21 (2 *μ*g/ml) for 72 h. Beta-cell proliferation was expressed as % Ki-67-positive beta cells. ***P<0.001 *versus* vehicle; ^††^*P*<0.01 *versus* GH (*n*=4). (**c**) Representative immunostaining of islets labeled for insulin (green), DAPI (blue) and Ki-67 (red). Scale bar=100 *μ*m. Data are mean±SE

Insulin gene expression and beta-cell proliferation were measured to further examine the effects of FGF21 on GH actions at the functional levels. As shown in Figure 5a, GH treatment (100 ng/ml, 72 h) increased insulin mRNA expression by ~2.5-fold in normal mouse islets, whereas co-treatment with FGF21 inhibited GH's effect dose dependently. In addition, GH (100 ng/ml, 72 h) increased beta-cell proliferation in normal mouse islets, whereas FGF21 (2 *μ*g/ml) reduced GH's effect (Figures 5b and c; vehicle=0.25±0.06; GH=0.93±0.11; GH+FGF21=0.48±0.11% Ki-67-positive beta cells).

### FGF21 inhibits islet GH signaling via modulating the expression of PPAR*γ* and CIS

**Figure 6:**
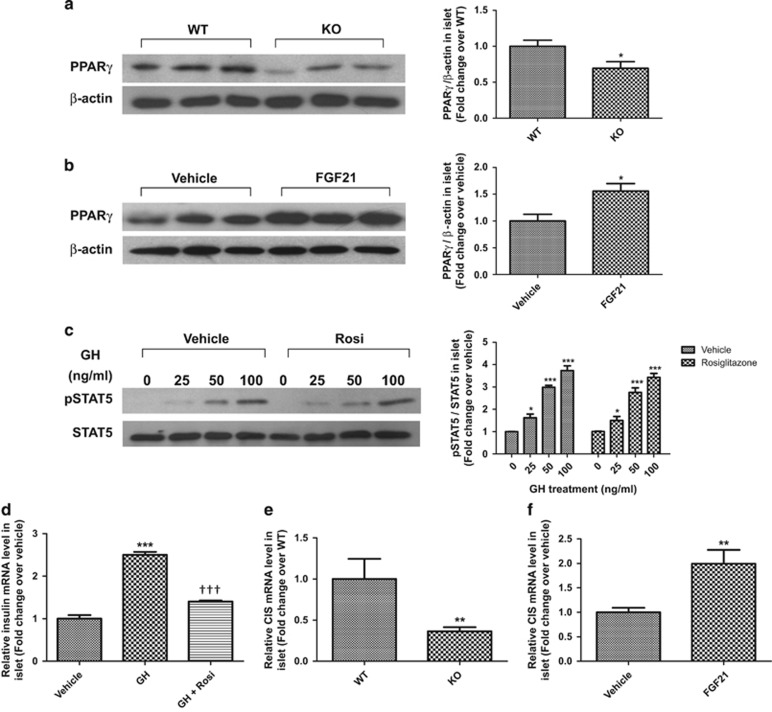
FGF21 inhibits islet GH signaling via induction of PPAR*γ* and CIS expression. (**a**) Protein levels of PPAR*γ* in FGF21-KO and WT mouse islets. *P<0.05 *versus* WT (*n*=3). (**b**) Protein levels of PPAR*γ* in normal mouse islets treated with FGF21 (2 *μ*g/ml) for 72 h. *P<0.05 *versus* vehicle (*n*=3). (**c**) Phosphorylated and total STAT5 in normal mouse islets pre-treated with rosiglitazone (Rosi; 20 *μ*mol/l) for 72 h, then stimulated with GH for 15 min *P<0.05, ***P<0.001 *versus* 0 ng/ml (*n*=3). (**d**) Insulin mRNA levels of normal mouse islets treated with vehicle, GH (100 ng/ml) and with or without rosiglitazone (Rosi; 20 *μ*mol/l) for 72 h. ***P<0.001 *versus* vehicle; ^†††^*P*<0.001 *versus* GH (*n*=4). (**e**) CIS mRNA levels in FGF21-KO and WT mouse islets. **P<0.01 *versus* WT (*n*=4–6 mice/batch; three batches). (**f**) CIS mRNA levels in normal mouse islets treated with FGF21 (2 *μ*g/ml) for 72 h. **P<0.01 *versus* vehicle (*n*=4). Data are mean±SE

To study the role of PPAR*γ* in the inhibitory effects of FGF21 on GH signaling in islets, PPAR*γ* expression was compared between FGF21-KO and WT mouse islets. As shown in Figures 6a and b, PPAR*γ* expression was reduced by 30% in FGF21-KO mouse islets, whereas FGF21 (2 *μ*g/ml, 72 h) increased PPAR*γ* expression in normal mouse islets by ~1.5-fold. Rosiglitazone (20 *μ*mol/l), a PPAR*γ* agonist, did not block GH-induced STAT5 phosphorylation (Figure 6c), but did reverse GH-induced insulin mRNA expression in normal mouse islets (Figure 6d). The role of CIS in FGF21-induced inhibition of GH signaling was also investigated. Notably, CIS mRNA expression was reduced by 63% in FGF21-KO mouse islets (Figure 6e), whereas FGF21 increased CIS expression by approximately twofold in normal mouse islets (Figure 6f). In addition, expression of SOCS1 and SOCS3 was unaltered by FGF21 treatment (Supplementary Figures S5A and B), whereas recombinant adiponectin (1 *μ*g/ml) did not affect PPAR*γ* expression and GH-induced STAT5 phosphorylation in islets (Supplementary Figures S5C and D), suggesting that these molecules were not involved in FGF21's actions.

### FGF21-KO mouse islets exhibit impaired glucose-stimulated insulin secretion (GSIS)

**Figure 7:**
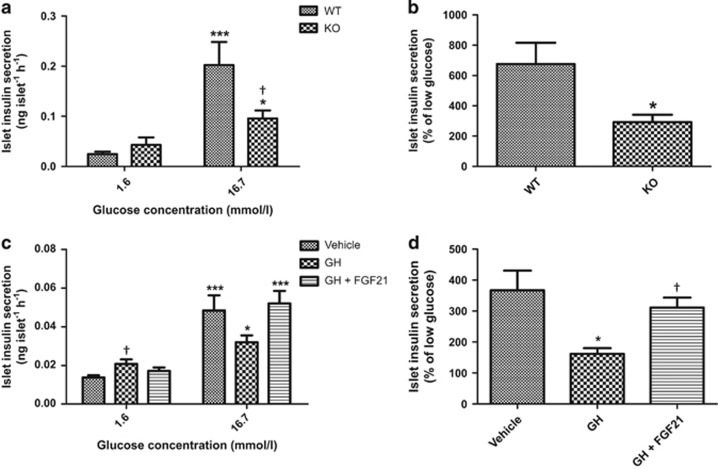
FGF21-KO mice display impaired GSIS. (**a**) Islet insulin secretion in WT and FGF21-KO mice. *P<0.05, ***P<0.001 *versus* 1.6 mmol/l; ^†^*P*<0.05 *versus* WT 16.7 mmol/l. (**b**) Islet insulin secretion was expressed as % of low glucose. *P<0.05 *versus* WT (*n*=4–6 mice/batch; four batches). (**c**) Islet insulin secretion in normal mouse islets pre-treated with GH (100 ng/ml) with or without FGF21 (2 *μ*g/ml) for 72 h. *P<0.05, ***P<0.001 *versus* 1.6 mmol/l; ^†^*P*<0.05 *versus* vehicle 1.6 mmol/l. (**d**) Islet insulin secretion was expressed as % of low glucose. *P<0.05 *versus* vehicle; ^†^*P*<0.05 *versus* GH (*n*=4). Data are mean±SE

For islet insulin-secreting capacity, high-glucose challenge induced insulin secretion in both WT and FGF21-KO mouse islets (Figure 7a). However, the insulin secretion upon glucose challenge was significantly reduced in FGF21-KO mouse islets, as shown by the reduced % of low glucose (Figure 7b; WT=676.23±140.4; KO=292.36±48.66% of low glucose). To test whether enhanced GH signaling impairs GSIS in FGF21-KO mice, isolated islets from normal mice were treated with GH, with or without FGF21 prior to GSIS assessment. As shown in Figure 7c, GH (100 ng/ml, 72 h) increased basal insulin secretion (in 1.6 mmol/l low glucose) as compared with vehicle. Insulin release in response to high glucose was, however, reduced by GH treatment, but co-treatment with FGF21 (2 *μ*g/ml) reversed the inhibitory effect of GH on GSIS (Figure 7d; Vehicle=366.78±63.87; GH=161.76±18.61; GH+FGF21=311.44±32.29% of low glucose).

### Inhibition of GH signaling in FGF21-KO mouse islets downregulates insulin expression and rescues the impaired GSIS

**Figure 8:**
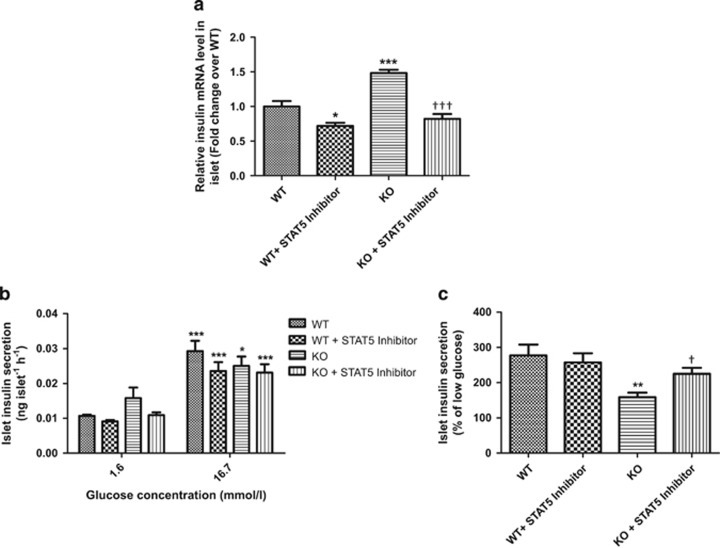
Inhibition of GH signaling rescues the phenotypes of FGF21-KO mouse islets. Isolated islets from WT and KO mice were treated with or without STAT5 inhibitor (10 *μ*g/ml) for 72 h. (**a**) Islet insulin mRNA levels. *P<0.05, ***P<0.001 *versus* WT; ^†††^*P*<0.001 *versus* KO (*n*=3). (**b**) Islet insulin secretion. *P<0.05, ***P<0.001 *versus* 1.6 mmol/l. (**c**) Islet insulin secretion was expressed as % of low glucose. **P<0.01 *versus* WT; ^†^*P*<0.05 *versus* KO (*n*=4). Data are mean±SE

To further confirm the role of GH signaling in the phenotypes of FGF21-KO mouse islets, insulin expression and GSIS were measured after inhibition of STAT5. As shown in Figure 8a, inhibition of basal GH signaling in WT mouse islets reduced insulin expression, whereas the increased insulin mRNA level in FGF21-KO mouse islets was almost totally reversed by STAT5 inhibitor (10 *μ*g/ml, 72 h). For insulin-secreting ability, STAT5 inhibition did not significantly alter GSIS in WT mouse islets but partially rescued the impaired GSIS in FGF21-KO mouse islets (Figures 8b and c; WT=277.18±31.00; WT+STAT% inhibitor=257.44±25.92; KO=159.13±12.80; KO+STAT5 inhibitor=225.05±17.19% of low glucose), indicating an improved response to the high-glucose challenge.

## Discussion

The present study is the first to report the modulatory action of FGF21, probably via GH signaling, in the pathogenesis of insulin resistance, and its regulatory role in pancreatic islet response to insulin resistance. FGF21 treatments improve glycemic control, insulin sensitivity and islet function in various animal models.^3, 4, 5, 6, 7^ In corroboration, clinical studies have also revealed associations between serum FGF21 levels and parameters of glucose metabolism.^29, 30^ However, most previous *in vivo* studies on FGF21 were based on the gain-of-function approaches and whether endogenous FGF21 is an obligatory metabolic regulator remains unclear. In this study, we found that loss of FGF21 *in vivo* led to increased insulin resistance without changes in glycemia. On the other hand, FGF21 deficiency induced islet hyperplasia and hyperinsulinemia as compensatory responses, which were due, at least in part, to the removal of inhibitory effects of FGF21 on islet GH signaling.

It has been known that glucose is a direct inducer of beta-cell hyperplasia.^31^ However, in many situations with insulin resistance, both insulin synthesis and beta-cell proliferation are increased without overt changes in glycemia.^32^ Among circulating hormones or growth factors, GH is well documented as antagonizing the metabolic actions of insulin^14, 15, 33^ and increasing insulin resistance,^17, 18^ as well as being implicated in the islet hyperplasia seen with insulin resistance.^32^ In the current study, we have demonstrated that FGF21 inhibited GH signaling downstream of the GHR through reduction of GH-induced JAK2 and STAT5 phosphorylation, insulin expression, and beta-cell proliferation in pancreatic islets. The loss of FGF21 led to increased islet GH sensitivity without changing circulating GH levels, as evidenced by enhanced GH-induced JAK2 and STAT5 phosphorylation at the molecular level; this in turn resulted in increased beta-cell proliferation, islet size and insulin production, the well-known effects of GH on islets.^19, 20, 21^ Islet cell mass is generally governed by the balance between cell proliferation and apoptosis; however, our study has shown no change in islet apoptosis in FGF21-deficient state, suggesting that islet hyperplasia in FGF21-KO mice was largely due to enhanced cell proliferation.

As FGF21 and GH share other target organs, such as the adipose tissue, a major site for insulin, increased insulin resistance under FGF21 deficiency may reflect altered GH signaling in such organs, whilst concurrent compensatory islet responses would tend to maintain euglycemia in FGF21-KO mice. Several major endocrine tissues (e.g., liver, adipose tissue and pancreatic islets) have been shown to be resistant to FGF21 in obese diabetic mice,^8, 34^ hence the use of global KO mice might mimic these *in vivo* diabetic conditions, when particularly attempting to explore the roles of FGF21 during T2DM pathogenesis. On the other hand, our *ex vivo* data have clearly shown that FGF21 directly inhibited GH signaling in isolated islets, as shown by the inhibition on GH-induced JAK2 and STAT5 phosphorylation, as well as GH-induced insulin expression and beta-cell proliferation, which were consistent with the observations in FGF21-KO mice. These data also support our hypothesis that loss of FGF21 would directly regulate islet cell growth and function *in vivo*. Noticeably, a previous study by Wente *et al.*^7^ has shown that FGF21 preserves islets and beta cells in *db/db* diabetic mice through reduction of islet glucolipotoxicity and apoptosis, whereas FGF21 does not affect islet cell proliferation. Together with the findings in this study, these results suggest that FGF21 may only inhibit beta-cell expansion induced by excess growth stimuli, but not in *db/db* mouse islets where most of the beta cells are exhausted. Moreover, systemic administration of FGF21 improves insulin sensitivity and relieves hyperinsulinemia in *ob/ob*, *db/db* mice, diet-induced obese mice and diabetic monkeys,^3, 4, 5, 6, 7^ which are consistent with our data that loss of FGF21 induces insulin resistance and hyperinsulinemia. These findings suggest that FGF21 may act as a positive regulator to maintain normal islet cell mass in different physiological conditions, as well as to maintain normal insulin sensitivity and insulin secretion.

The next issue to address is how FGF21 inhibits islet GH signaling. In this regard, we have shown that FGF21 inhibited GH-induced signaling, probably through the effects on PPAR*γ* and CIS, independent of GHR expression. PPAR*γ* is a member of the nuclear hormone receptor family, well known to regulate diverse biological processes, including glucose and lipid homeostasis.^35^ Specifically, targeted elimination of PPAR*γ* in beta cells is associated with islet hyperplasia,^36^ whereas activated PPAR*γ* inhibits GH signaling.^13^ We here report that FGF21 increased islet PPAR*γ* expression while PPAR*γ* was downregulated in FGF21-deficient islets, which were in line with previous findings that FGF21 promotes PPAR*γ* activity and expression in adipose tissue.^37, 38^ Rosiglitazone-induced PPAR*γ* activation reversed GH-induced islet insulin expression without affecting GH-induced STAT5 phosphorylation, which were consistent with previous work that PPAR*γ* inhibits transcriptional activity of STAT5 but not tyrosine phosphorylation.^13^ Therefore, the inhibitory effects of FGF21 on islet JAK2 and STAT5 phosphorylation should be mediated by other pathways. GH signaling is also modulated by SOCS protein and CIS, which specifically inhibit JAK2 and STAT5 phosphorylation.^11, 12^ Our findings of reduced CIS mRNA levels in FGF21-KO mouse islets, and the FGF21-induced increase in CIS expression in normal mouse islets, suggest that FGF21 may inhibit GH signaling through the induction of both PPAR*γ* and CIS expression, so that the reduction of PPAR*γ* and CIS levels in FGF21-KO mouse islets could lead to enhanced GH sensitivity. Recent studies have elucidated a regulatory mechanism between FGF21, PPAR*γ* and adiponectin in adipose tissue in which adiponectin is a mediator of some specific metabolic actions of FGF21.^39, 40^ Our *ex vivo* studies have demonstrated that direct FGF21 treatments in isolated islets enhanced PPAR*γ* expression and reduced GH signaling. To determine whether adiponectin is involved in the action of FGF21 in islets *in vivo*, we treated isolated islets with recombinant adiponectin and found that adiponectin did not alter PPAR*γ* expression and GH-induced signaling, suggesting that the effects of FGF21 on islet GH signaling is independent of adiponectin.

FGF21-KO mice exhibited distorted islet architecture and reduced insulin-secreting capacity during glucose challenge both *in vivo* and *ex vivo*, suggestive of islet dysfunction. The molecular mechanism(s) whereby normal spatial distribution of cell types in islets is maintained remains largely unknown. As abnormal islet structure is often accompanied by impaired GSIS, as observed in Glut2-deficient and glucokinase-deficient mice,^41, 42^ it is plausible to speculate that abnormal islet architecture itself contributes to impaired GSIS. In addition, FGF21-KO mice displayed increased alpha-cell population even though no difference was found in mRNA or serum levels of glucagon. As GH does not directly stimulate alpha-cell proliferation,^20^ increased alpha-cell population may be caused by increased insulin, which is known to promote alpha-cell proliferation^43^ but to inhibit both gene transcription and secretion of glucagon.^44, 45, 46^ Thus, GH may directly and indirectly increase islet cell population, which contributes to islet hyperplasia in FGF21-KO mice. Dual effects of GH on islet insulin secretion have been previously reported, with GH stimulating insulin release directly but inhibiting glucose-induced insulin release in isolated islets.^47^ In corroboration, our results showed that both GH-treated normal mouse islets and FGF21-KO mouse islets displayed enhanced basal insulin secretion but impaired insulin secretion in response to glucose challenge, which were consistent with the *in vivo* data that FGF21-KO mice exhibited elevated basal serum insulin but reduced response to glucose administration during the glucose tolerance test. These results demonstrate that enhanced GH sensitivity in FGF21-KO mouse islets altered both basal and stimulated insulin secretion.

In the present study, we found that FGF21 inhibited GH signaling in islets, reversed GH-induced insulin expression, beta-cell proliferation, and impaired GSIS. On the other hand, GH sensitivity was enhanced in FGF21-KO mouse islets while the increased insulin expression and impaired GSIS could be rescued by blockade of GH signaling. These data further suggest that those observed phenotypes in FGF21-KO mouse islets are due, at least in part, to the removal of FGF21 inhibition on islet GH signaling. Our findings thus demonstrate that FGF21 is a critical factor in the maintenance of insulin sensitivity, normal islet cell growth, insulin synthesis, and islet function.

In conclusion, our findings support that FGF21 is an obligatory metabolic regulator in pancreatic islets, which sheds new light into the pathophysiological role of FGF21 in insulin resistance and islet dysfunction.

## Materials and Methods

### Animal models

Male FGF21-KO and WT mice of C57BL/6J origin were prepared as we described previously.^27, 48, 49^ Male normal C57BL/6J mice were obtained from the Laboratory Animal Services Center of the Chinese University of Hong Kong. The experimental procedures were approved by the Animal Experimentation Ethics Committee of the Chinese University of Hong Kong (Ref. No. 12/062/MIS-5).

### *In vivo* glucose homeostasis

Glucose tolerance was assessed by an IPGTT. After 6 h fasting, mice were given 1.5 g/kg body weight of glucose (Sigma-Aldrich, St. Louis, MO, USA) by intraperitoneal injection and blood glucose was then measured. For the intraperitoneal insulin tolerance test, mice were injected with insulin (0.3 U/kg body weight; Eli Lilly and Company, Indianapolis, IN, USA) after 4 h fasting, and blood glucose levels were subsequently measured. Degrees of insulin resistance were estimated by HOMA-IR using the following equation: HOMA-IR=fasting serum insulin (mU/l) × fasting blood glucose (mmol/l)/22.4.

### Pancreatic islet isolation, primary culture, and treatments

Intact pancreatic islets were isolated from mice as previously described.^8^ Briefly, mouse pancreas was given intra-ductal injection of 0.3 mg/ml collagenase P (Roche, Mannheim, Germany) in Hanks' balanced salt solution (Sigma-Aldrich). The pancreas was removed and incubated in 37 °C for 15 min. After washing and gradient centrifugation, the islets were handpicked under a stereomicroscope. Isolated islets were then cultured overnight in RPMI-1640 medium (Life Technologies, Carlsbad, CA, USA) supplemented with 10% (vol/vol) bovine fetal serum (Gibco, Grand Island, NY, USA), 1% (vol/vol) penicillin, and streptomycin (Life Technologies). Islets were treated with recombinant FGF21 (University of Hong Kong; HKU, Hong Kong, China),^50^ recombinant GH (R&D Systems, Minneapolis, MN, USA), rosiglitazone (Sigma-Aldrich) or STAT5 inhibitor (Santa Cruz Biotechnology, Santa Cruz, CA, USA).

### Reverse transcription (RT)-PCR

Total RNA from islets was extracted using TRIzol reagent (Life Technologies) and subjected to reverse transcription (RT) using the cDNA Synthesis Kit (Bio-Rad, Munich, Germany). Gene expression was quantified by real-time PCR using SYBR Green Supermix (Bio-Rad) in a Thermal Cycler (Bio-Rad). Relative gene expression was analyzed by the 2^−ΔΔCt^ method, and normalized relative to glyceraldehyde 3-phosphate dehydrogenase (GAPDH).^8^ The primer sequences used were as follows: mouse GAPDH forward, 5′-GCACAGTCAAGGCCGAGAAT-3′ mouse GAPDH reverse, 5′-GCCTTCTCCATGGTGGTGAA-3′ mouse insulin forward, 5′-AGCGTGGCTTCTTCTACACAC-3′ mouse insulin reverse, 5′-CTGGTGCAGCACTGATCTACA-3′ mouse GHR forward, 5′-CTTCTCAAGGAAGGGAAGTTG-3′ mouse GHR reverse, 5′-GAATCATCATCCTTTGCTCCA-3′ mouse CIS forward, 5′-CGCAGCGGACAAAAGATTAGG-3′ mouse CIS reverse, 5′-GGACAAGATCCCTGTACGCAA-3′ mouse SOCS1 forward, 5′-CACCTTCTTGGTGCGCG-3′ mouse SOCS1 reverse, 5′-AAGCCATCTTCACGCTGAGC-3′ mouse SOCS3 forward, 5′-GCTCCAAAAGCGAGTACCAGC-3′ mouse SOCS3 reverse, 5′-AGTAGAATCCGCTCTCCTGCAG-3′.

### Western blotting

Islet protein was extracted using the CytoBuster Protein Extraction Reagent (Novagen, Darmstadt, Germany). Around 40–50 *μ*g proteins were loaded for each sample. Proteins were separated by SDS-PAGE, transferred to nitrocellulose membrane (Bio-Rad). After blocking, the membrane was probed with antibodies against the following proteins: *β*-actin, PPAR*γ* (Santa Cruz Biotechnology), phospho-JAK2 (Y1008), JAK2, phospho-STAT5 (Y694), STAT5 (Cell Signaling, Danvers, MA, USA). After washing, the membrane was probed with appropriate horseradish peroxide-conjugated secondary antibodies. Labeled protein bands were revealed by the ECL detection reagent (GE Healthcare, Piscataway, NJ, USA) on autoradiography films (Fuji Film, Tokyo, Japan). The western blot bands were quantitated with ImageJ software (National Institutes of Health, Bronx, NY, USA).

### Measurement of islet insulin release

Fifteen size-matched islets were pre-incubated in Krebs-Ringer bicarbonate buffer (KRBB; supplemented with 10 mmol/l HEPES and 2 mg/ml BSA) with 1.6 mmol/l glucose for 1.5 h before incubation in KRBB with 1.6 mmol/l glucose for 1 h, followed by incubation in KRBB containing 16.7 mmol/l glucose for an additional 1 h, as described previously.^51^ Buffer was collected to measure insulin release by an enzyme-linked immunosorbent assay (ELISA; HKU).^39^

### Measurement of serum hormones

Commercial ELISA kits were used to determine serum insulin (HKU) and glucagon (Sigma-Aldrich) according to the manufacturer's instructions.

### Pancreatic islet staining

Fresh pancreata or isolated islets were embedded and frozen. Cryostat sections were collected and fixed, as described previously.^51^ For hematoxylin–eosin staining, pancreatic sections were stained with hematoxylin (Sigma-Aldrich) and then counterstained with eosin (Sigma-Aldrich). After washing with water and graded dehydration using ethanol and xylene, slides were mounted with entellan (Merck, Darmstadt, Germany). For immunostaining, slides were incubated with guinea pig anti-insulin (Life Technologies) and rabbit anti-Ki-67 (Abcam, Cambridge, MA, USA) for the detection of beta-cell proliferation and incubated with rabbit anti-insulin (Santa Cruz Biotechnology) and mouse anti-glucagon (Abcam) for the assessment of islet morphology. After washing, slides were probed with appropriate fluorescent-conjugated secondary antibodies. After counterstaining with DAPI and washing, slides were mounted with VectaShield medium (Vector-Laboratories, Burlingame, CA, USA) before image acquirement. Digital images were acquired by a fluorescence microscope equipped with a DC200 digital camera (Leica Microsystems, Wetzlar, Germany) and were analyzed by Leica Qwin image analysis software (Leica Microsystems).

### Statistical analysis

Data were expressed as means±S.E. Comparisons between groups were analyzed by a two-tailed Student's *t-*test, or one-way analysis of variance, followed by Tukey's *post hoc* test, where *P*<0.05 was considered statistically significant.

## Abbreviations

CIS: cytokine-inducible SH-2 containing protein
ELISA: enzyme-linked immunosorbent assay
FGF: fibroblast growth factor
GAPDH: glyceraldehyde 3-phosphate dehydrogenase
GH: growth hormone
GHR: growth hormone receptor
GSIS: glucose-stimulated insulin secretion
HOMA-IR: homeostasis model assessment of insulin resistance
IPGTT: Intraperitoneal glucose tolerance test
JAK: janus kinases
KO: knockout
KRBB: Krebs-Ringer bicarbonate buffer
PPAR*γ*: peroxisome proliferator-activated receptor gamma
RT: reverse transcription
SOCS: suppressor of cytokine signaling
STAT: signal transducers and activators of transcription
T2DM: type 2 diabetes mellitus
WT: wild type
