# Supplementary material for: Loss of fibroblast growth factor 21 action induces insulin resistance, pancreatic islet hyperplasia and dysfunction in mice
Source: Cell Death Dis. 2015 Mar 26;6(3):e1707–. doi: 10.1038/cddis.2015.80 (PMC4385948; doi:10.1038/cddis.2015.80)
Supplement: Supplementary Figures [file cddis201580x1.pdf]

Supplementary figure

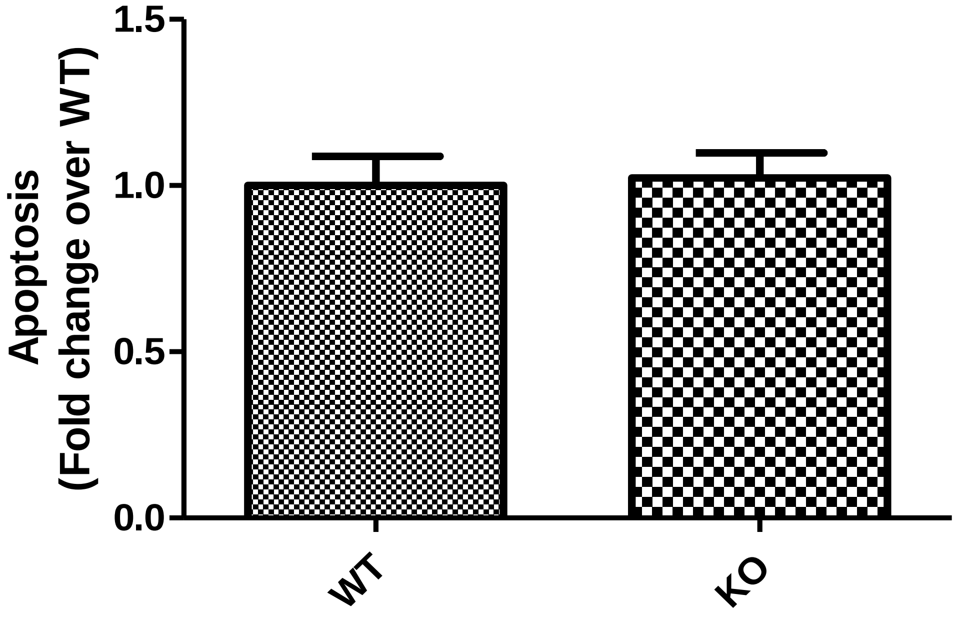

Supplementary figure S1

Supplementary figure

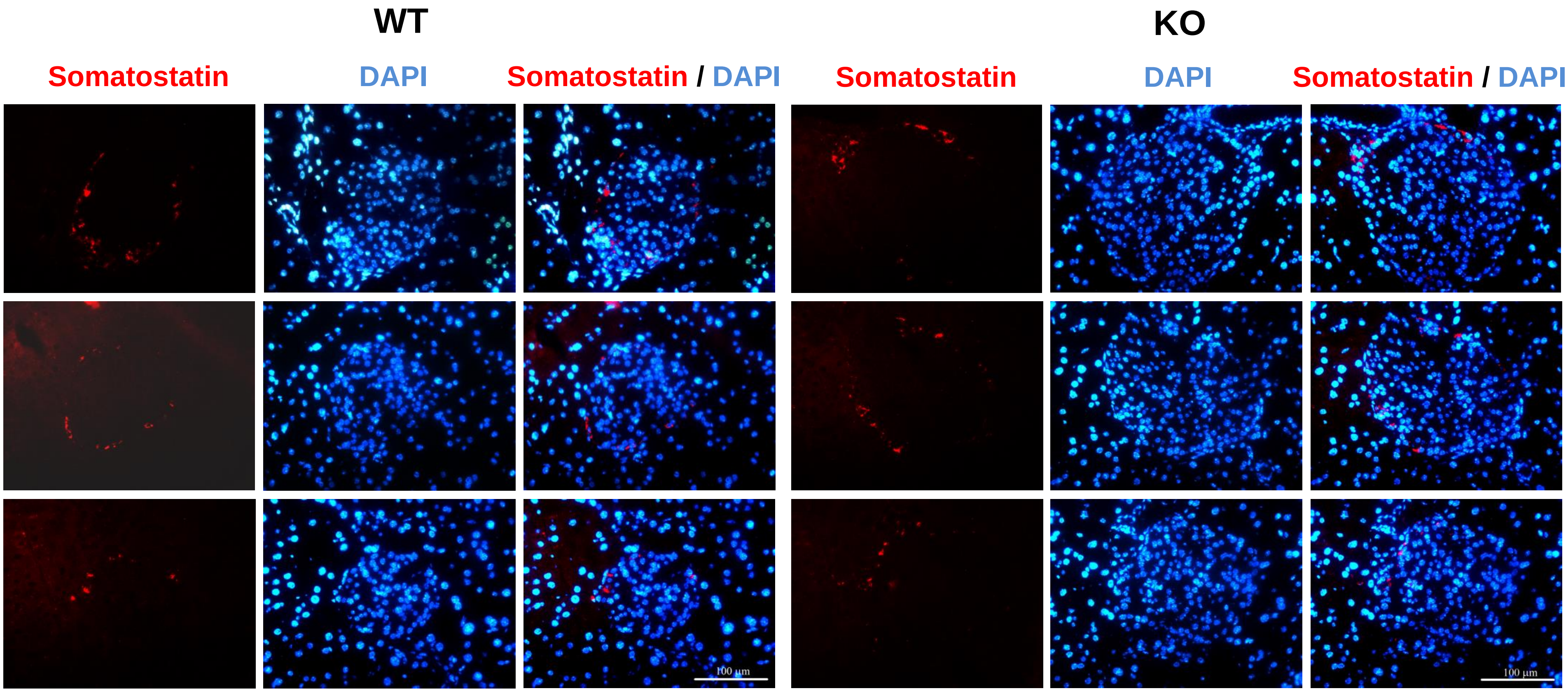

Supplementary figure S2

Supplementary figure

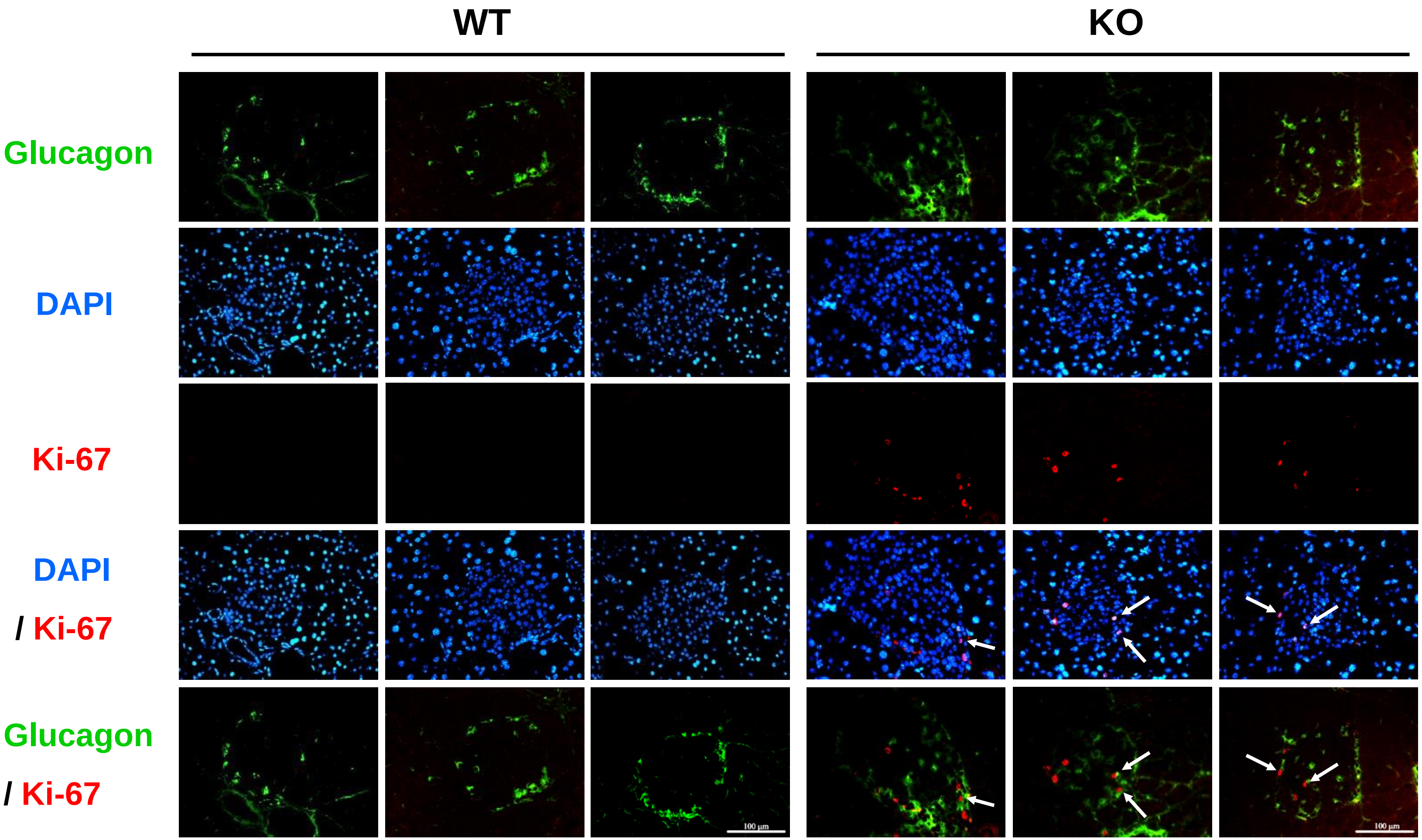

Supplementary figure S3

Supplementary figure

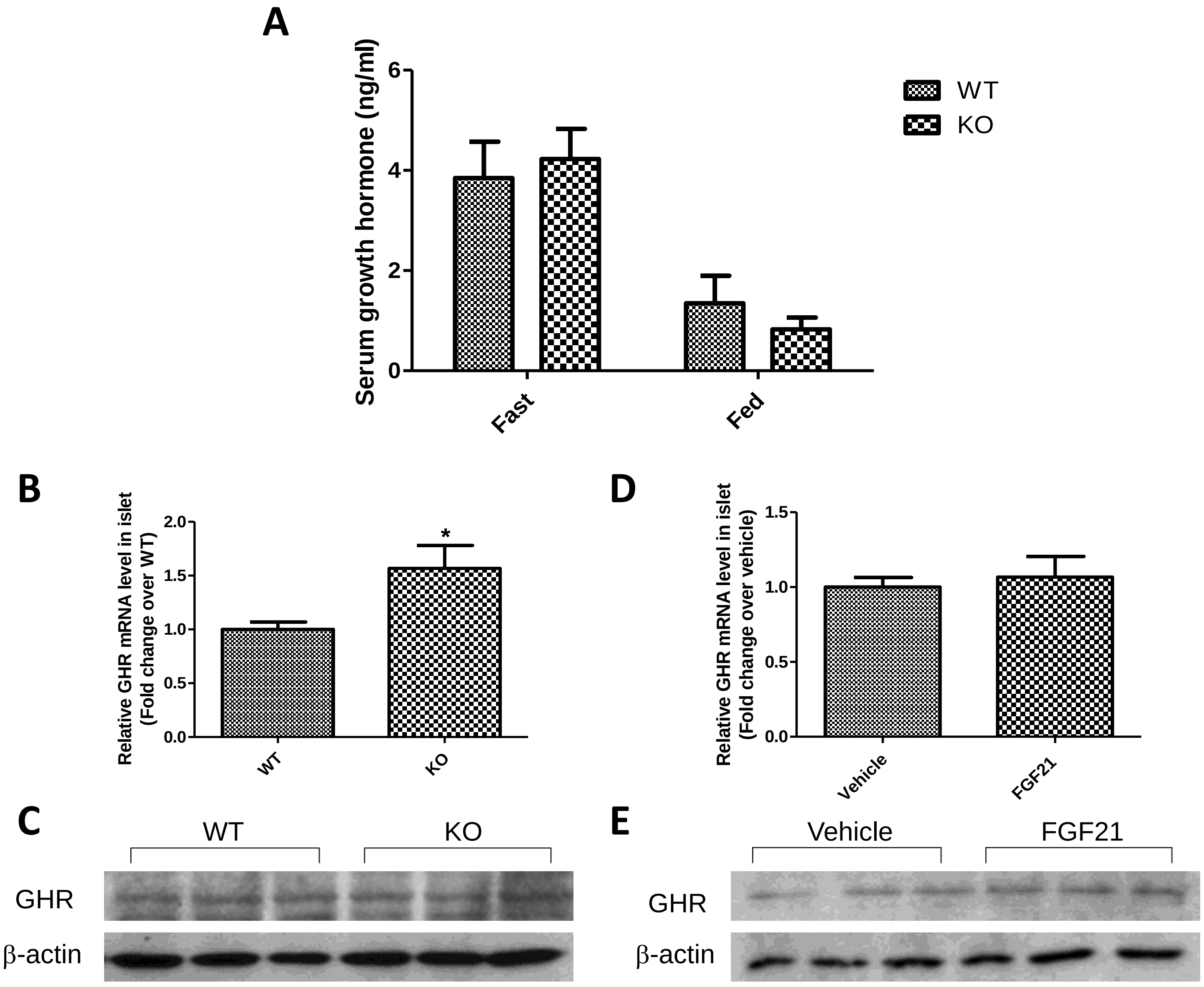

Supplementary figure S4

Supplementary figure

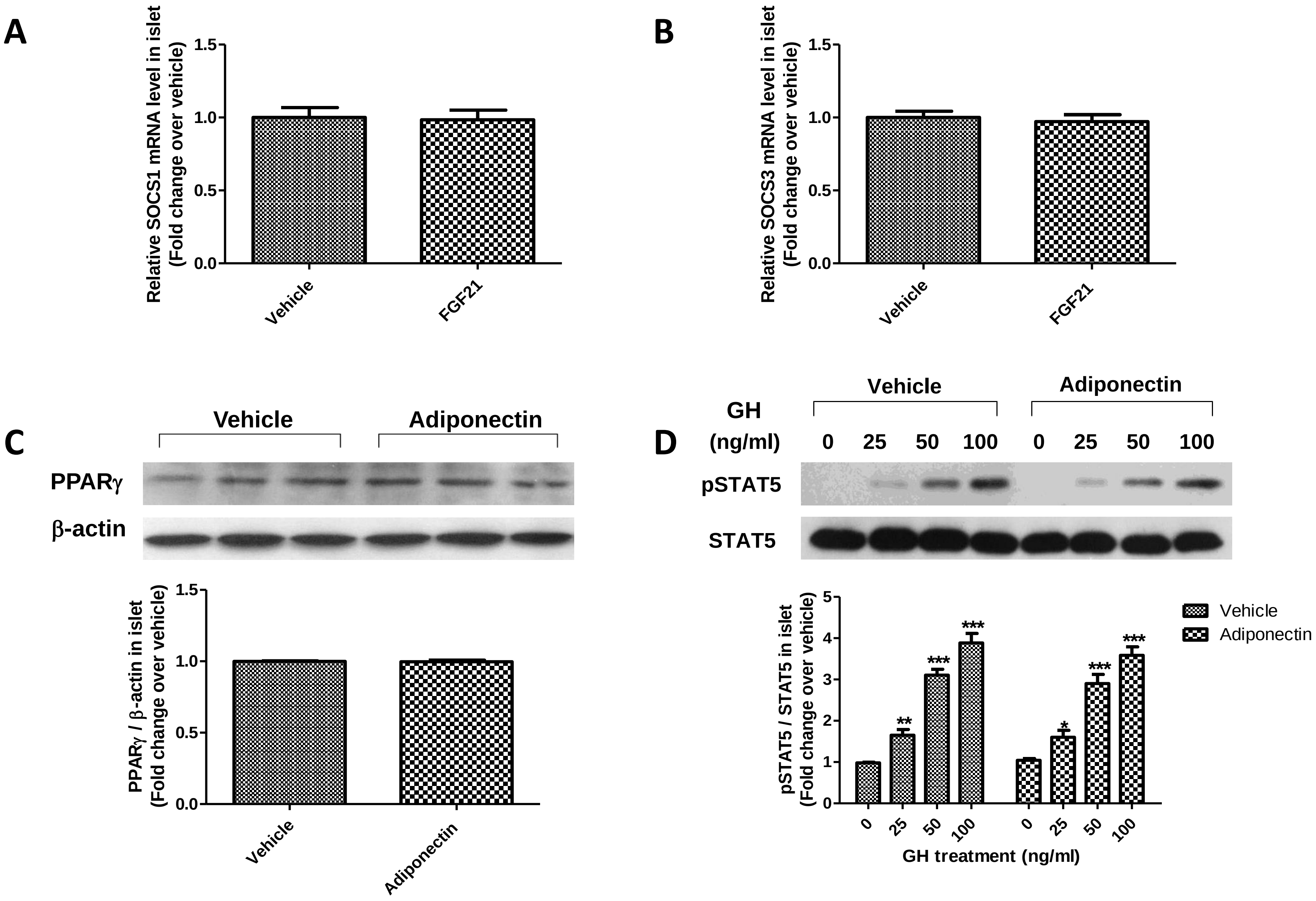

Supplementary figure S5
