# Supplementary material for: Loss of fibroblast growth factor 21 action induces insulin resistance, pancreatic islet hyperplasia and dysfunction in mice
Source: Cell Death Dis. 2015 Mar 26;6(3):e1707–. doi: 10.1038/cddis.2015.80 (PMC4385948; doi:10.1038/cddis.2015.80)
Supplement: Supplementary Figure Legends [file cddis201580x2.doc]

**Legends to Supplementary Figures**

**Supplementary Fig. S1** FGF21 deficiency does not affect islet apoptosis. Islet apoptosis was measured by Cell Death Detection ELISA plus kits (Roche, Mannheim, Germany) (*n*=4-6 mice/batch; 3 batches). Data are means ± SEs.

**Supplementary Fig. S2** FGF21 deficiency does not affect delta-cell distribution. Representative immunostaining of islets labeled for somatostatin (red) and DAPI (blue). Scale bar = 100 μm. (*n*=3 mice/batch; 3 batches).

**Supplementary Fig. S3** FGF21-KO mice display increased alpha-cell proliferation. Representative immunostaining of islets labeled for glucagon (green), DAPI (blue) and Ki-67 (red). Scale bar = 100 μm. (*n*=3 mice/batch; 3 batches).

**Supplementary Fig. S4** FGF21 does not affect GH production or the expression of GHR**.** (A) Serum GH levels were measured in WT and FGF21-KO mice in fed and overnight-fasted states by ELISA kits (Millipore, St Charles, MO, USA) (*n*=4-6 mice/batch; 3 batches). Islets were isolated from WT and FGF21-KO mice, total (B) RNA and (C) protein were extracted. Islets were isolated from normal mice and treated with FGF21 (2 μg/ml) for 72 h, total (D) RNA and (E) protein were extracted. GHR mRNA expression was detected using specific primers by quantitative RT-PCR; while protein expression was detected using specific antibody (Novus Biologicals, Littleton, CO, USA) by western blotting. **P*<0.05 vs. WT (*n*=3). Data are means ± SEs.

**Supplementary Fig. S5** SOCS1, SOCS3 and adiponectin may not be involved in the actions of FGF21 in islets. Islets were isolated from normal mice and treated with FGF21 (2 μg/ml) for 72 h. mRNA expression of (A) SOCS1 and (B) SOCS3 was detected using specific primers by quantitative RT-PCR (*n*=4). Islets were isolated from normal mice and treated with recombinant adiponectin (HKU; 1 μg/ml) for 72 h, (C) PPARγ expression and (D) GH-induced STAT5 phosphorylation (100 ng/ml, 15 min) were determined. **P*<0.05; ***P*<0.01; ****P*<0.001 vs. 0 ng/ml (*n*=3). Data are means ± SEs.
